# 1,3-Dipolar [3 + 2] cycloaddition reactions of *N*,*C*,*C*-trisubstituted nitrones with ring-acceptor methylenecyclopropanes: a computational study

**DOI:** 10.1186/s40064-016-3758-0

**Published:** 2016-12-05

**Authors:** Mary Mensah, Evans Elikem Amepetey, Richard Tia, Evans Adei

**Affiliations:** Department of Chemistry, Kwame Nkrumah University of Science and Technology, PMB University Post Office, Kumasi, Ghana

**Keywords:** Methylenecyclopropanes, Nitrones, Regioselectivity, Density functional theory, Spirocyclopropanes

## Abstract

**Background:**

1,3-Dipolar [3 + 2]-cycloaddition of nitrones to the carbon–carbon double bonds of methylenecyclopropanes yields a mixture of regioisomeric 4- and 5-isoxazolidines. The mechanisms of the reactions of *N*,*C*,*C*-trisubstituted nitrones with ring acceptor substituted dimethyl methylenecyclopropanes-1,2-dicarboxylate and aryl methylidene cyclopropanes-1,1-dicarboxylate have been investigated with the Becke 3-Parameter Lee–Yang–Par exchange–correlation functional, a Hartree–Fock DFT hybrid functional, to delineate the factors responsible for the regioselectivity of these class of reactions.

**Findings:**

The energetics of the reaction of the phenyl-substituted nitrone with unsubstituted methylenecyclopropane indicate that the formation of the 5-spirocyclopropane is favored over the 4-spirocyclopropane kinetically and thermodynamically. However, the energetics of the reaction of the same phenyl nitrone with vicinal ester (–CO_2_CH_3_)-substituted methylenecyclopropane show an inversion in the regioselectivity favoring the formation of the 4-regioisomer over the 5-regioisomer. For the reactions of *N*,*C*,*C*-trisubstituted nitrone with vicinal ester (–CO_2_CH_3_)-substituted methylenecyclopropane (–R_1_=H, –R_2_=Ph and –R_1_=CH_3_ and –R_2_=CO_2_CH_3_) and geminal ester (–CO_2_CH_3_)-substituted methylenecyclopropane (R_3_=H, R_4_=H; R_3_=OCH_3_, R_4_=CH_3_; and R_3_=H, R_4_=Cl), the energetics indicate that the 5-spirocyclopropane is favored over the 4-spirocyclopropane. The calculations also indicate that electron-donating groups increase regioselectivity of the 5-regioisomers over the 4-regioisomers.

**Conclusion:**

The regioselectivity of these reactions is determined by both electronic and steric factors. The pathways with the lower activation barrier leads to the more stable regioisomer in all cases, implying that the pathways that are kinetically favored are also thermodynamically favored. However, it is also clear from the energetics that these reactions are not reversible and are therefore under kinetic control. Therefore the selectivity of the reactions is governed solely by the difference in activation barriers leading to the two isomers and not in any way by the thermodynamic stability of the isomers formed.

## Background

The development of 1,3-dipolar cycloaddition reactions has in recent years entered a new stage as control of the regiochemistry in the addition step is now the major challenge. 1,3-Dipolar [3 + 2]-cycloaddition of nitrones to the carbon–carbon double bonds of methylenecyclopropanes yields a mixture of regioisomeric 4- and 5-isoxazolidines that are very useful due to their versatility, stereochemistry and their biological activities (Li et al. 2010). This mixture of products was observed in the chemistry of 5-spirocyclopropane isoxazolidines; these polar intermediates can lead to completely new processes with the selective and very efficient formation of new tetrahydro-1*H*-indolizin-5-ones (Cordero et al. 2004). Regioselectivity of 1,3-dipolar cycloaddition reactions in general is determined by electronic structure of reactants, steric factors or combination of both (Jones and Martin 2002). The isoxazolidine cycloadduct contains up to three new chiral centers and, as with other 1,3-dipoles, the highly ordered transition state often allows the regio- and stereo-chemical preference of a given nitrone to be predicted (Ess and Houk 2008).

Merino et al. (2003) performed a theoretical study at the DFT B3LYP/6-31G(d) theory level on the 1,3-dipolar cycloaddition reactions of *C*-(methoxycarbonyl)-*N*-methyl nitrone with methyl acrylate and vinyl acetate with the aim of defining the preferred approaches for cycloaddition reactions of nitrones and to introduce additional details in the commonly accepted general model of 1,3-dipolar cycloaddition of nitrones. They concluded that a theoretical preference for 3,5-regioisomers is observed, with the *trans* adduct being 1.0 kcal/mol more stable than the *cis* adduct. Diev et al. (2009) reported from their experimental studies that in reactions between acceptor ring-substituted methylenecyclopropanes and *C*,*N*-diarylnitrones, thermally stable regioisomeric 4-spirocyclopropane-isoxazolidines are formed instead of regioisomeric 5-spirocyclopropane-isoxazolidines which are observed for unsubstituted or alkyl-substituted methylenecyclopropanes. In 2012, Molchanov et al. conducted an experimental study on the 1,3-dipolar cycloaddition reaction of *C*,*C*-disubstituted ketonitrones with acceptor methylenecyclopropanes with the aim of inverting the regioselectivity of the 1,3-dipolar cycloaddition reaction with the formation of 5-spirocyclopropane isoxazolidine. *N*,*C*,*C*-trisubstituted nitrones were reacted with ring acceptor substituted dimethyl methylenecyclopropanes-1,2-dicarboxylate or arylmethylidenecyclopropane-1,1-dicarboxylates. The initial slow step in the studied reactions is a normal 1,3-dipolar cycloaddition of methylenecyclopropanes to nitrones to form spirocyclopropane-isoxazolidine cycloadducts. These cycloadducts are thermally unstable and undergo a thermally induced Brandi–Guarna rearrangement. This shows that the cycloadduct formed could not be isolated and regiochemical outcome is not certain. Hence, there is the need to carry out a detailed theoretical study to fully map out the mechanism of the reaction and ascertain the factors responsible for the regioselectivity of these class of reactions. The aim of this work is therefore to carry out a theoretical study on the reactions between *N*,*C*,*C*-trisubstituted nitrones with ring acceptor substituted dimethyl methylenecyclopropanes-1,2-dicarboxylate and aryl methylidene cyclopropanes-1,1-dicarboxylate to determine the regioselectivity of the reaction and explore the effects of substituents on the energetics of the reaction. The results of this study would help delineate the factors controlling the regiochemical outcome of these reactions.

## Methods

All the calculations were performed with the Spartan (Wavefunction 2010) computational chemistry package developed by Wavefunction, Inc, versions 2008V1.2.0 and 2010V1.2.0, using the Becke-three-parameter Lee–Yang–Parr (B3LYP) hybrid exchange-correlational functional (Becke 1988, 1996; Lee et al. 1988; Vosko et al. 1980) and the 6-31G* basis set.

Molecules were constructed with Spartan’s graphical user interface and minimized interactively using the MMFF force field. All structural optimizations were done without symmetry restrictions. Full frequency calculations were performed to verify that the correct stationary points were located. Minima, representing reactants, intermediates and products were shown to have a Hessian matrix with all positive eigen-values, characterized by all real vibrational frequencies.

Input structures for transition state optimizations were obtained by first constraining specific internal coordinates along the reaction coordinates while the fully optimizing the remaining internal coordinates. The approximate transition state structures obtained this way were then submitted for transition state calculations. All transition state structures were subjected to full frequency calculations to ensure that they have a Hessian matrix with a single negative eigen-value, characterized by an imaginary vibrational frequency along the reaction coordinate. An intrinsic reaction coordinate (IRC) calculation was performed to ensure that transition state structures located smoothly connect the reactants and products along the reaction coordinate (Ahmed et al. 2016).

## Results and discussion

### Reaction between *N*-phenyl substituted nitrone with unsubstituted methylenecyclopropane

**Scheme 1:**
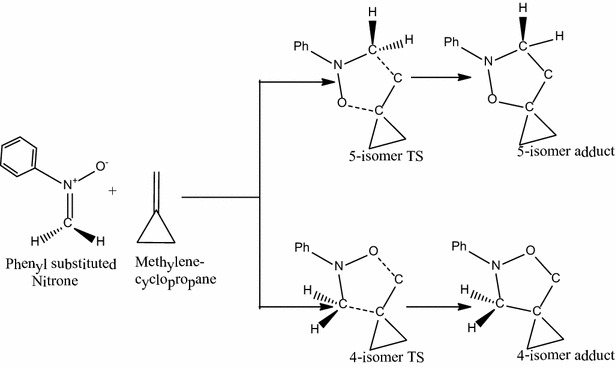
Proposed mechanism for the reaction between *N*-phenyl substituted nitrone and unsubstituted methylenecyclopropane

The reaction of phenyl substituted nitrone with unsubstituted methylenecyclopropane (i.e. R_1_=R_2_=H; Scheme 1) forms two possible transition state structures, 5-isomer transition state and 4-isomer transition states, leading to the two regioisomeric products.

The C–C and C–O forming bond lengths have been computed as 2.20 and 2.31 Å respectively for the 4-isomer transition state and 2.23 and 2.24 Å for the 5-isomer transition state respectively. The activation energy through the 4-isomer transition state is 11.8 kcal/mol whereas that through the 5-isomer transition state is 9.8 kcal/mol. Thus in this reaction, the 5-isomer transition state is 2.0 kcal/mol lower in energy than that of the 4-isomer transition state, favoring the formation of the 5-regioisomer kinetically. Both reactions are exothermic, with reaction energies of −37.8 and −39.6 kcal/mol respectively for the formation of the 4- and 5-isomers. Thus the 5-isomer adduct (**A1**) being more stable than the 4-isomer adduct (**A2**), which is consistent with the work of Molchanov et al. (2012). This reaction is not reversible and is therefore under kinetic control. Therefore, the selectivity of the reaction is governed solely by the difference in activation barriers between the two pathways and not by the stability of the products formed.

### Reactions of *N*,*C*,*C*-trisubstituted nitrones with ring acceptor substituted dimethyl methylenecyclopropane-1,2-dicarboxylate

**Scheme 2:**
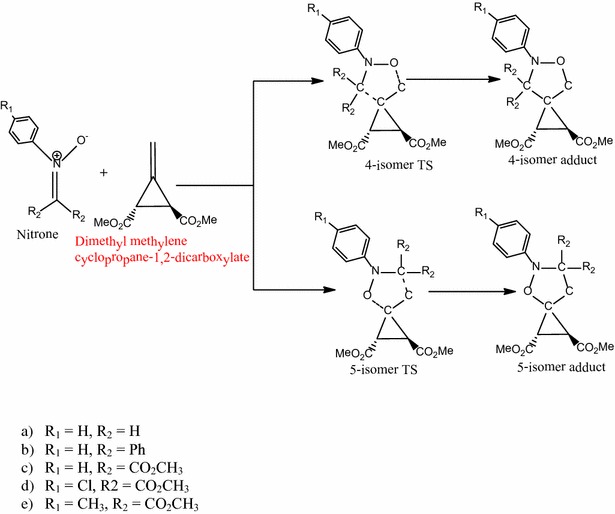
Proposed mechanism for the reaction between *N*,*C*,*C*-trisubstituted nitrones and dimethyl methylenecyclopropanes-1,2-dicarboxylate

Table 1 shows a comparison of the energetics of the 1,3-dipolar cycloaddition reactions of *N*-trisubstituted nitrone with vicinal ester (–CO_2_CH_3_)-substituted methylenecyclopropanes (Scheme 2).

**Table 1:** Energetics of the 1,3-dipolar cycloaddition reactions of *N*,*C*,*C*-trisubstituted nitrone with ring acceptor substituted dimethyl methylenecyclopropane-1,2-dicarboxylates

Substituents	Activation energy (kcal/mol)		Reaction energy (kcal/mol)	
	4-isomer	5-isomer	4-isomer	5-isomer
R_1_=–H, R_2_=–H	+8.5	+11.7	−40.7	−32.5
R_1_=–H, R_2_=–Ph	+30.3	+16.9	−9.1	−17.9
R_1_=–H, R_2_=–CO_2_CH_3_	+16.0	+13.6	−21.5	−36.7
R_1_=–Cl, R_2_=–CO_2_CH_3_	+15.6	+14.1	−22.7	−38.1
R_1_=–CH_3_, R_2_=CO_2_CH_3_	+16.0	+13.0	−21.1	−36.3

The reaction follows a concerted, pericyclic pathway along two possible transition states, 5-isomer transition state and 4-isomer transition state.

All the reactions are asynchronous with the formation of the 5-isomer being more asynchronous than the formation of 4-isomer in all reactions. As with the reaction of the *N*-phenyl substituted nitrone with unsubstituted methylenecyclopropane, the energetics show that the cycloaddition is not reversible and is under kinetic control. The 4-isomer adduct is shown to be favored over the 5-isomer adduct by 3.2 kcal/mol in the reaction with both R_1_=R_2_=H whereas the 5-isomer adduct is favored by 13.4, 2.4, 1.5 and 3.0 kcal/mol over the 4-isomer adduct in the reaction with R_1_=–H, R_2_=–Ph; R_1_=–H, R_2_=–CO_2_CH_3_; R_1_=–Cl, R_2_=–CO_2_CH_3_; and R_1_=–CH_3_, R_2_=–CO_2_CH_3_ as substituents.

In the reaction with R_1_=R_2_=H, the C–C and C–O forming bond lengths are 2.26 and 2.23 Å respectively for the 4-isomer transition state and 2.21 and 2.23 Å for the 5-isomer transition state respectively. The activation energy through the 4-isomer transition state is 8.5 kcal/mol whereas that through the 5-isomer transition state is 11.7 kcal/mol. Thus kinetically the 4-isomer is favored over the 5-isomer. The formation of the 4-isomer product has a reaction energy of −40.73 kcal/mol while that of the 5-isomer has an energy of −32.5 kcal/mol. The reaction is thus exothermic, with the 4-isomer adduct (**A4**) being more stable than the 5-isomer adduct (**A3**). Since these reactions are not reversible and therefore cannot be under thermodynamic control, the selectivity is controlled by the difference in activation barriers alone.

In the reaction with R_1_=–H, R_2_=–Ph, the C–C and C–O forming bond lengths are 2.60 and 1.82 Å respectively for the 4-isomer transition state and 2.32 and 2.00 Å for the 5-isomer transition state respectively. The activation energy for the formation of the 4-isomer is 30.3 kcal/mol whereas that for the formation of the 5-isomer is 16.9 kcal/mol, showing that the 5-isomer is kinetically favored over the 4-isomer transition state. The 4-isomer has a reaction energy of −9.1 kcal/mol and the 5-isomer has an energy of −17.9 kcal/mol. The reaction is thus exothermic with the 5-isomer adduct (**A5**) being more stable than the 4-isomer adduct (**A6**). The phenyl group is seen to have a deactivating effect on the reaction along both pathways, but it makes the formation of the 5-isomer more feasible than the formation of the 4-isomer and also increases the selectivity of the preferred pathway.

In the reaction with R_1_=–H, R_2_=–CO_2_Me, the C–C and C–O forming bond lengths are 2.40 and 2.02 Å respectively for the 4-isomer transition state and 2.2 and 2.14 Å for the 5-isomer transition state respectively. The activation energy through the 4-isomer transition state is 16.0 kcal/mol whereas that through the 5-isomer transition state is 13.6 kcal/mol showing that the 5-isomer is kinetically favored over the 4-isomer. The 4-isomer (**A8**) has a reaction energy of −21.5 kcal/mol and the 5-isomer (**A7**) has an energy of −36.7. The selectivity towards the formation of the 5-isomer is lower in the reaction of the methylcarboxylate-substituted reactant compared to the phenyl-substituted reactant even though both reactions are more favorable with the methylcarboxylate substituent than with the phenyl substituent.

In the reaction with R_1_=–Cl, R_2_=–CO_2_CH_3_, the C–C and C–O forming bond lengths are 2.40 and 2.03 Å respectively for the 4-isomer transition state and 2.26 and 2.16 Å for the 5-isomer transition state respectively. The 5-isomer is shown to be kinetically favored over the 4-isomer by 1.5 kcal/mol. The formation of the 4-isomer (**A10**) has a reaction energy of −22.7 kcal/mol and of the 5-isomer (**A9**) has an energy of −38.1 kcal/mol. The presence of the –Cl group on the benzene ring on the nitrone decreases the activation barrier of the 4-isomer by 0.4 kcal/mol and increases the activation barrier of the 5-isomer by 0.5 kcal/mol thus reducing the regioselectivity towards the formation of the 5-isomer adduct. However thermodynamically, the stability of the adducts formed are increased.

In the reaction with R_1_=–CH_3_, R_2_=–CO_2_CH_3_, the C–C and C–O forming bond lengths are 2.40 and 2.01 Å respectively for the 4-isomer transition state and 2.23 and 2.11 Å for the 5-isomer transition state respectively. The activation energy for the formation of the 4-isomer is 16.0 kcal/mol whereas that for the 5-isomer is 13.0 kcal/mol showing that the 5-isomer is kinetically favored over the 4-isomer. The 4-isomer (**A12)** has reaction energy of −21.1 kcal/mol and the 5-isomer (**A11**) has an energy of −36.3 kcal/mol. The presence of electron donating methyl (–CH_3_) group on the nitrone results in higher regioselectivity towards the formation of the 5-isomer relative to the nitrones with substituents –Cl and –H).

### The reaction of *N*-phenyl-*C*,*C*-dimethylcarboxylate substituted Nitrone with methylenecyclopropane-1,1-dicarboxylate

**Scheme 3:**
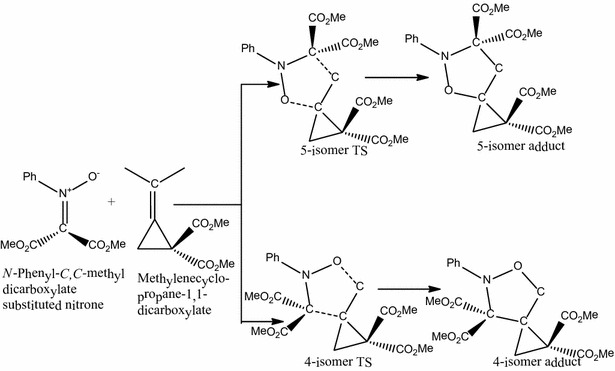
Proposed mechanism for the reaction between *N*-phenyl-*C*,*C*-dimethylcarboxylate substituted nitrone with methylenecyclopropane-1,1-dicarboxylate

The reaction of *N*-phenyl-*C*,*C*-dimethylcarboxylate substituted nitrone with methylenecyclopropane-1,1-dicarboxylate (Scheme 3) follows a concerted, pericyclic pathway and produces two transition state structures; 5-isomer transition state and 4-isomer transition state.

The C–C and C–O bond lengths are 2.23 and 2.24 Å respectively for the 5-isomer transition state structure while for the 4-isomer transition state structure they are 2.20 and 2.31 Å respectively. The activation energy through the 5-isomer transition state is 9.8 kcal/mol while that through the 4-isomer transition state is 11.8 kcal/mol, showing that the 5-isomer transition state is favored over the 4-isomer transition state. Thus the formation of the 5-isomer is favored since the reaction is under kinetic control. The 4-isomer (**A14**) has a reaction energy of −37.8 kcal/mol and the 5-isomer (**A13**) has an energy of −39.6 which makes it 1.8 kcal/mol more stable than the 4-isomer.

### Reaction between *N*-phenyl-*C*,*C*-dimethylcarboxylate substituted nitrone with aryl methylidenecyclopropane-1,1-dicarboxylate

**Scheme 4:**
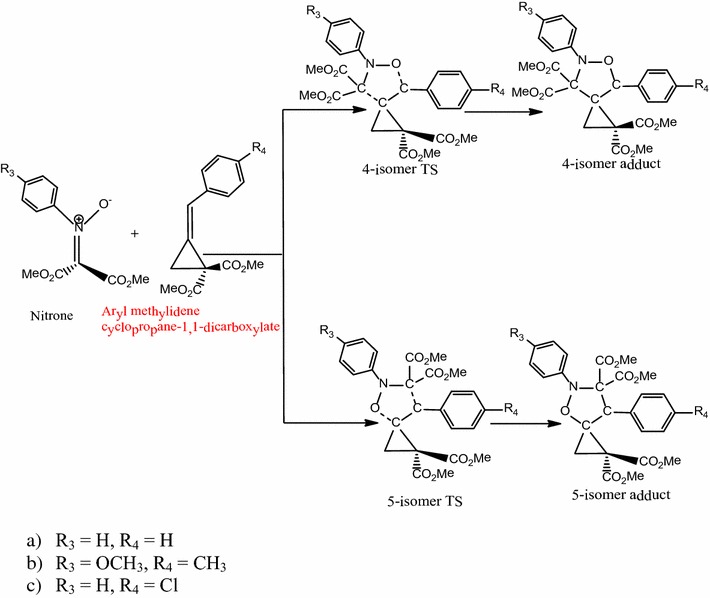
Proposed mechanism for the reaction between *N*,*C*,*C*-trisubstituted nitrones and aryl methylidene cyclopropanes-1,1-dicarboxylate

Table 2 shows a comparison of the energetics of the 1,3-dipolar cycloaddition reactions of *N*,*C*,*C*-trisubstituted nitrone with geminal ester (–CO_2_CH_3_)-substituted methylenecyclopropanes (Scheme 4). The reaction follows a concerted, pericyclic pathway and shows two possible transition state structures, 5-isomer transition state and 4-isomer transition state. All the reactions are asynchronous with the formation of the 5-isomer being more asynchronous than the formation of the 4-isomer in all reactions. The formation of the 5-isomer is more favorable than that of the 4-isomer in all cases since all the reactions are under kinetic control.

**Table 2:** Energetics of the reaction with *N*,*C*,*C*-trisubstituted nitrones with aryl methylidenecyclopropane-1,1-dicarboxylates

Substituents	Activation energy (kcal/mol)		Reaction energy (kcal/mol)	
	4-isomer	5-isomer	4-isomer	5-isomer
R_3_=–H, R_4_=–H	+28.9	+16.6	−10.3	−20.9
R_3_=–OCH_3_, R_4_=–CH_3_	+29.7	+17.6	−10.0	−20.4
R_3_=–H, R_4_=–Cl	+29.1	+16.2	−10.4	−22.0

In the reaction with R_3_=R_4_=–H, the C–C and C–O bond lengths are 2.43 and 2.0 Å respectively for the 5-isomer transition state while for the 4-isomer transition state they are 2.33 and 2.14 Å respectively. The activation energy through the 5-isomer transition state is 16.6 kcal/mol while that through the 4-isomer transition state is 28.9 kcal/mol. The formation of the 5-isomer is kinetically favored over the 4-isomer while the 5-isomer (**A15**) is more stable than the 4-isomer (**A16**). This indicates that the effects of the sterically bulky ester and phenyl groups on both reactants increase regioselectivity towards the formation of the 5-isomer.

In the reaction with R_3_=–OCH_3_, R_4_=–CH_3_, the C–C and C–O forming bond lengths are 2.40 and 2.0 Å respectively for the 5-isomer transition state while for the 4-isomer transition state they are 2.31 and 2.13 Å respectively. The 5-isomer is shown to be kinetically favored over the 4-isomer by 12.1 kcal/mol. The formation of the 5-isomer (**A17**) has a reaction energy of −20.4 kcal/mol while the formation of the 4-isomer (**A18**) has an energy of −10.0 kcal/mol. The energetics show that the electron-donating (–OCH_3_ and –CH_3_) groups increase the activation barrier by 0.8 and 1.0 kcal/mol respectively for the 4-isomer and 5-isomer and the adducts formed are less stable relative to the reaction with R_3_=R_4_=–H.

For reaction with R_3_=–H, R_4_=–Cl, the C–C and C–O forming bond lengths are 2.42 and 2.0 Å respectively for the 5-isomer transition state while that for the 4-isomer transition state are 2.31 and 2.14 Å respectively. The activation energy through the 5-isomer transition state is 16.2 kcal/mol while that through the 4-isomer transition state is 29.1 kcal/mol, showing that the 5-isomer is kinetically favored over the 4-isomer while the 5-isomer (**A19**) is more stable than the 4-isomer (**A20**) by 11.6 kcal/mol. The energetics indicate that electron-withdrawing (–Cl) group decreases the activation barrier by 1.4 and 0.6 kcal/mol for the 5-isomer and 4-isomer respectively but the regioselectivity towards the 5-isomer increases. Thermodynamically, the adducts formed are more stable relative to the reaction with R_3_=R_4_=–H.

## Conclusion

The results of the study show that in the 1,3-dipolar [3 + 2] cycloaddition reactions between *N*,*C*,*C*-trisubstituted nitrone with dimethyl methylenecyclopropane-1,2-dicarboxylate, the formation of the 4-isomer is favored over that of the 5-isomer when R_1_=R_2_=H. However, the selectivity switches towards the 5-isomer when the substituents R_1_=H, R_2_=Ph; R_1_=H, R_2_=CO_2_CH_3_; R_1_=Cl, R_2_=CO_2_CH_3_; R_1_=CH_3_, R_2_=CO_2_CH_3_ are used. Also, in reactions between *N*,*C*,*C*-trisubstituted nitrone with arylmethylidenecyclopropane-1,1-dicarboxylate with *para* substituents the 5-isomer is favored both kinetically and thermodynamically over 4-isomer in all cases. Electron withdrawing groups on both nitrone and methylenecyclopropane tend to increase regioselectivity towards the formation of the 5-isomer, even though we are at present not able to assign a reason for this trend.

**Fig. 1:**
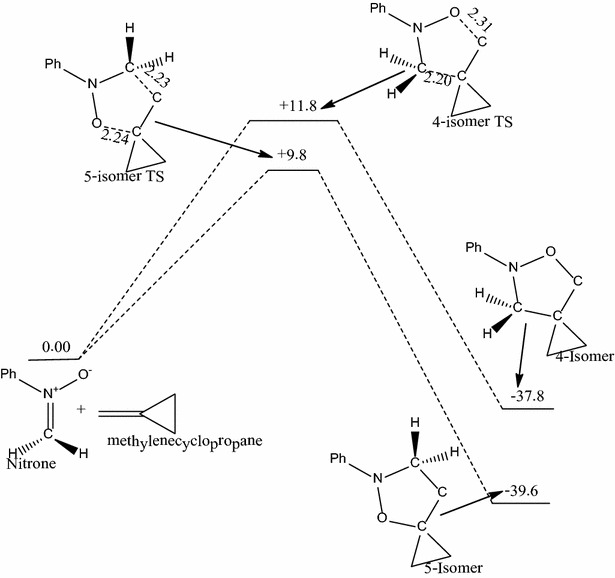
Energetics of the reaction of *N*-phenyl substituted nitrone with unsubstituted methylenecyclopropane. Energies in kcal/mol and bond lengths in Å

**Fig. 2:**
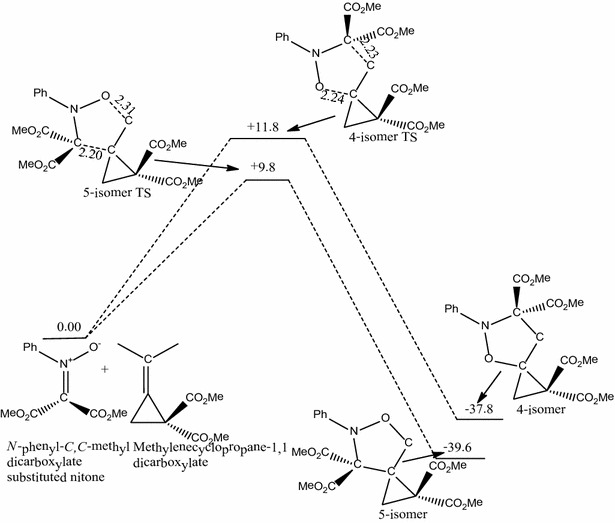
Energetics for the reaction of *N*-phenyl-*C*,*C*-dimethylcarboxylate substituted nitrone with methylenecyclopropane-1,1-dicarboxylate. Energies in kcal/mol and bond lengths in Å

 The results show that the pathways with the lower activation barrier leads to the more stable regioisomer in all cases, implying that the pathways that are kinetically favored are also thermodynamically favored. However, it is also clear from the energetics that these reactions are not reversible and are therefore under kinetic control. Therefore the selectivity of the reactions is governed solely by the difference in activation barriers leading to the two isomers and not in any way by the thermodynamic stability of the isomers formed (Figs. 1, 2).
